# Greater increase in internal carotid artery shear rate during aerobic interval compared to continuous exercise in healthy adult men

**DOI:** 10.14814/phy2.14705

**Published:** 2021-01-19

**Authors:** Shigehiko Ogoh, Takuro Washio, Kazuya Suzuki, Motoyuki Iemitsu, Takeshi Hashimoto, Erika Iwamoto, Damian M. Bailey

**Affiliations:** ^1^ Department of Biomedical Engineering Toyo University Kawagoe‐Shi Japan; ^2^ Neurovascular Research Laboratory University of South Wales Pontypridd UK; ^3^ Research Fellow of Japan Society for the Promotion of Science Tokyo Japan; ^4^ Faculty of Sport and Health Science Ritsumeikan University Shiga Japan; ^5^ School of Health Sciences Sapporo Medical University Sapporo Japan

**Keywords:** cerebral blood flow, endothelial function, humans, internal carotid artery, shear stress

## Abstract

Interval exercise has been determined to be more effective than continuous exercise for achieving improvement in the cardiovascular function of individuals suffering from cardiovascular disease. However, whether interval exercise improves the cerebrovascular function remains unclear. As per our hypothesis, interval exercise induces a higher cerebrovascular shear rate (SR) than continuous exercise. In this study, 11 adult men randomly performed continuous exercise for 12 min or work‐equivalent (57.6 kJ/exercise session) interval exercise of semi‐recumbent cycling. The SR in the internal carotid artery (ICA) represents an index of the cerebrovascular SR, which was measured during both the exercises using Doppler ultrasonography. Both the aerobic exercise modes increased the ICA SR. Moreover, the average ICA SR of the interval exercise for the final 4 min of exercise or 2 min of recovery was significantly higher than that for continuous exercise (exercise, 351 ± 75 vs. 330 ± 61/s, *p* = .038; recovery, 327 ± 86 vs. 290 ± 56/s, *p* = .014). To our knowledge, this is the first study to show that aerobic interval exercise increased the ICA SR more than equivalent work volume of aerobic continuous exercise. Thus, aerobic interval exercise may be more effective at stimulating the cerebrovasculature, resulting in greater improvements in cerebrovascular function as compared to continuous aerobic exercise in healthy adult men. These findings provide some important information that would help enhance exercise therapy programs for patients with arteriosclerosis, especially in the cerebral circulation.

## Graphical abstract

Internal carotid artery image and cerebrovascular parameters during continuous and interval exercise by Doppler ultrasonography.

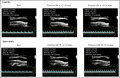

## 1 INTRODUCTION

An increase in physical activity has been determined to decrease the risk of cardiovascular (Booth & Lees, 2007) and cerebrovascular diseases (Fletcher, 1994). Both aerobic and resistance interval exercises are known to be more effective than continuous exercise for improving cardiorespiratory fitness, which is a strong predictor of mortality (Blair et al., 1996; Jung et al., 2019; Tjonna et al., 2008), and cardiovascular function in patients with cardiovascular diseases (Francois et al., 2016; Karstoft et al., 2014; Mitranun et al., 2014; Tjonna et al., 2008). Thus, recently, both aerobic and resistance interval exercises have been recommended to lower these risks (Calverley et al., 2020; Lucas et al., 2015). In fact, patients with brain‐related pathologies, such as stroke, have already begun using aerobic interval exercise protocol for rehabilitation (Boyne et al., 2013). However, the physiological evidence of this recommendation has not been fully established, especially for the brain. Exercise‐induced decrease in these risks is suggested to be primarily related to improved systemic endothelial function (Joyner & Green, 2009). In fact, research has shown that aerobic interval training was found to be superior to continuous moderate exercise for enhancing endothelial function in the systemic vasculature in patients with metabolic syndrome patients (Tjonna et al., 2008). Endothelial cell phenotype associated with endothelial function is regulated by hemodynamic forces, notably shear stress, generated by luminal blood flow (Chatzizisis et al., 2007; Davies, 2009). It is noteworthy that blood velocity and shear rate (SR) associated with the flow pattern contribute to endothelial function (Chatzizisis et al., 2007; Thijssen et al., 2009), which can be independently modified as a function of the exercise mode. As per a recent study (Lyall et al., 2019), aerobic interval exercise can cause a different pattern of SR in the systemic vasculature as compared to an equivalent work volume of aerobic continuous exercise; interval exercise caused a greater increase in the oscillatory SR as compared to continuous exercise. However, to our knowledge, no study has examined the impact of interval exercise, especially with respect to blood velocity and SR profile, on the cerebral vasculature. It is noteworthy that in the systemic vasculature, acute leg exercise increases the brachial retrograde SR, irrespective of low exercise workload (Simmons et al., 2011), and increases in retrograde SR augments oscillatory SR (Lyall et al., 2019). In contrast, retrograde SR has not been identified in the cerebrovasculature (Rodrigues et al., 2020). Therefore, exercise‐induced changes in the SR are likely to be site‐specific. In agreement with this finding, previous trials have shown that hypoxia‐induced increase was observed in the mean SR in the cerebral vasculature, but not in the systemic vasculature (Iwamoto et al., 2020; Tremblay et al., 2018).

In the systemic vasculature, although exercise‐induced increases in antegrade SR are greater during continuous exercise than during interval exercise, the ratio of retrograde SR to total SR (i.e., oscillatory shear index) is higher during interval exercise than during continuous exercise (Lyall et al., 2019), indicating that the effect of retrograde SR on the mean SR may be higher during interval exercise. Retrograde SR has a harmful effect on the endothelial function (Simmons et al., 2011; Thijssen et al., 2009). Despite an increase in the total SR, indeed, interval exercise did not improve the systemic endothelial function more than the continuous exercise (Lyall et al., 2019). In contrast, in the cerebral vasculature, given the lack of retrograde flow that contributes to SR larger, especially during interval exercise, the effect of interval exercise on the net increase in SR (i.e., mean SR) is expected to be greater than that of continuous exercise. However, an effect of the different exercise modes on cerebral SR needs to be examined. This important information indicates the possibility that interval exercise rather than continuous exercise improves cerebral endothelial function for decreasing the risk of cerebrovascular diseases (Calverley et al., 2020). Further, cerebrovascular improvement is caused largely by cerebral circulation compared with systemic vascular improvement. Thus, in this present study, we hypothesized that compared to continuous exercise, interval exercise would be associated with elevated SR in the cerebral vasculature. In order to test this hypothesis, we examined the SR in the internal carotid artery (ICA) as an index of regional cerebrovascular SR (Carter et al., 2016; Smith et al., 2019) in adult healthy men during aerobic interval exercise and compared it to an equivalent work volume of aerobic continuous exercise.

## 2 METHODS

### 2.1 Ethics

The study was approved by the Human Subjects Committee of the Toyo University (TU2018‐001). Before the experiment, each participant provided informed written consent and visited the laboratory to familiarize the techniques and procedures involved in the study. All the procedures conformed to the standards of the *Declaration of Helsinki*; however, this study was not registered in a database.

### 2.2 Participants

Eleven healthy adult men (age; 21.4 ± 0.8 years, height; 1.73 ± 0.03 m, body mass; 62.1 ± 6.4 kg, mean ± standard deviation [SD]) were enrolled in this study. None of the participants had any known cardiovascular or pulmonary disorders and were not using any prescribed or over‐the‐counter medications or taking food supplements including antioxidant vitamins. Moreover, the participants did not engage in endurance training on a regular basis (< 5 h/week). When visiting the laboratory, the participants were requested to abstain from caffeinated beverages for 12 h and strenuous physical activity and alcohol for at least 24 h before the day of the experiment. In addition, the experiment was performed after at least 3 h postprandial, following a low fat/high protein meal.

### 2.3 Design

**FIGURE 1:**
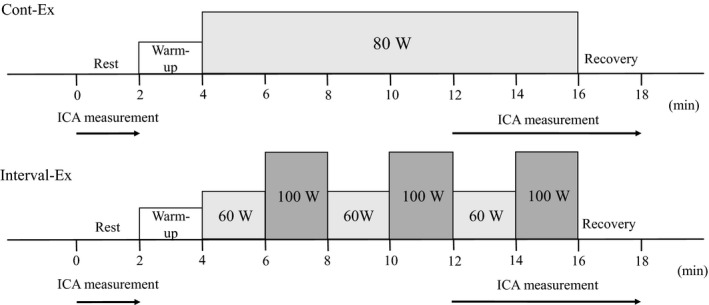
Experimental protocol of continuous exercise (Cont‐Ex, the upper panel) and an equivalent work‐equivalent interval exercise (Interval‐Ex, the bottom panel) in semi‐recumbent cycling

Each participant was randomly assigned to perform either of the following two exercise trials: aerobic continuous exercise (Cont‐Ex) or work‐equivalent (57.6 kJ/exercise session) aerobic interval exercise (Interval‐Ex). Subjects performed leg cycling using the aero‐bike (Aerobike 75XL III, Combi) at 60 rpm in the semi‐recumbent with both arms extended laterally and placed on the examination table. The cycling revolutions were checked throughout the exercise. Cycling was preceded by 15 min of rest and a standardized warm‐up at 40 Watts (W). Each trial was separated by 30 min to allow for full hemodynamic recovery. During the Cont‐Ex, the participants performed continuous cycling at 80 W for 12 min (80 W × 12 min × 60 s = 57.6 kJ). During the Interval‐Ex, the participants performed three bouts of interval cycling {each consisting of 2 min at 60 W and 2 min at 100 W, [(60 W × 2 min + 100 W × 2 min) × 3 bouts × 60 s = 57.6 kJ]} that were equivalent workload to Cont‐Ex (Figure 1).

### 2.4 Measurements

All studies were performed in a room at a constant temperature (23°C–24°C) and quiet environment. The participants were instrumented for measures of heart rate (HR) using a lead II electrocardiogram (bedside monitor, BMS‐3400; Nihon Kohden). Beat‐to‐beat arterial blood pressure (BP) was monitored continuously via finger photoplethysmography from the middle finger of the left hand (Finapres Medical Systems) to determine the systolic and diastolic blood pressure (SBP and DBP) as well as mean arterial pressure (MAP). Stroke volume (SV) was determined from the BP waveform using the Modelflow software program, incorporating sex, age, stature, and body mass (Beat Scope1.1; Finapres Medical Systems BV). End‐tidal partial pressure of carbon dioxide (P_ET_CO_2_), minute ventilation (V̇_E_), respiratory rate (RR), and oxygen uptake (V̇O_2_) were sampled from a leak‐free mask and measured using a gas analyzer (AE‐310S; Minato Medical Science Co.).

**FIGURE 2:**
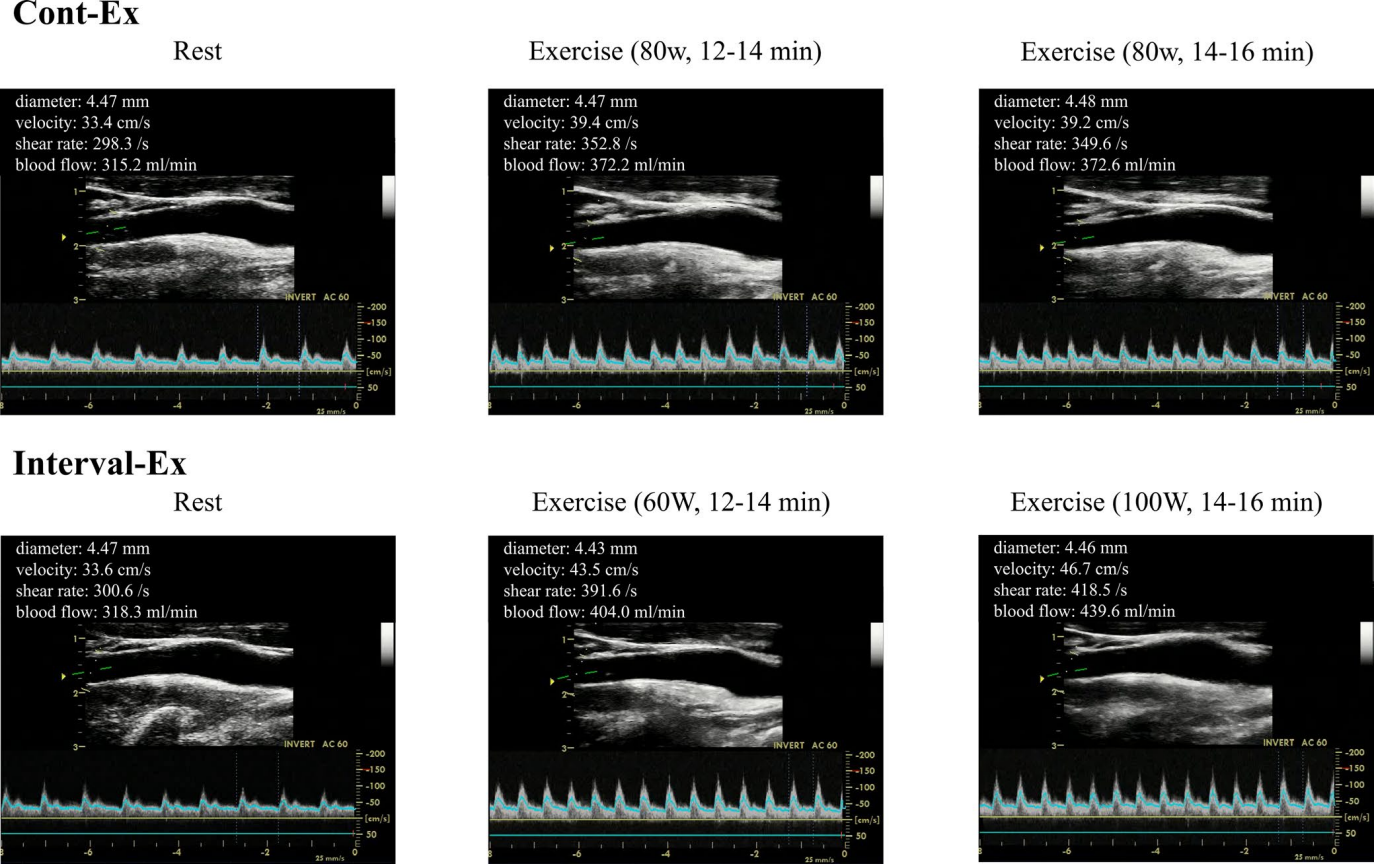
Internal carotid artery image and cerebrovascular parameters using Doppler ultrasonography

The ICA blood flow on the right side of the neck was measured using duplex ultrasonography (Vivid‐i; GE Healthcare) equipped with 13‐MHz linear transducers (Figure 2). The ICA blood flow was measured approximately 1.0–1.5 cm cranial to the carotid bifurcation. The time‐averaged mean blood velocity was obtained in the pulsed‐wave mode in a longitudinal plane. Stable probe position was ensured, such that the insonation angle remained constant (≤60°), and that the sample volume was positioned in the center of the vessel and adjusted to cover its width.

### 2.5 Data processing

**FIGURE 3:**
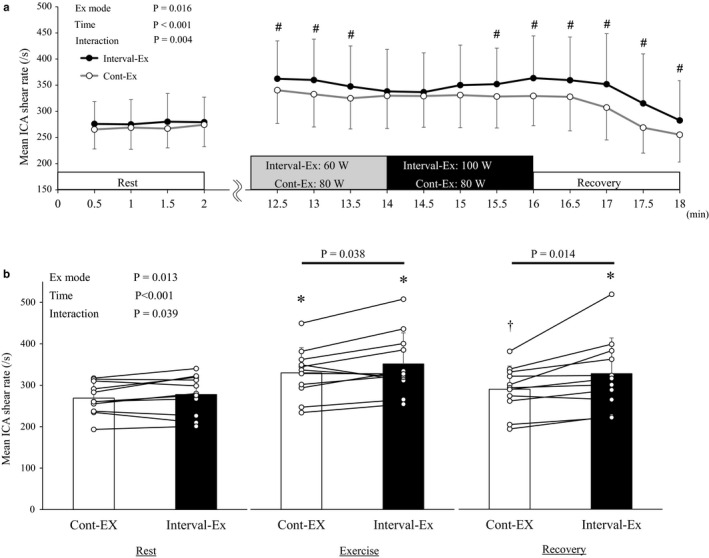
(a) Changes in the shear rate (SR) of the internal carotid artery (ICA) at the last bout of Interval (Interval‐Ex) or Continuous exercise (Cont‐Ex) and its recovery (n = 11). ^#^ *p* < .05 different from Cont‐Ex. These variables during rest, exercise, and recovery were averaged for 30 s. Data are presented as means ± SD. (b) Mean SR of the ICA for 2 min at rest (Rest), over the final 4 min during each exercise (Exercise), and for 2 min after stopping the exercise (Recovery) (n = 11). The bar and lines represent group average and individual values. ^*^ *p* < .05 different from Rest, ^†^ *p* < .05 different from Exercise. Group data are presented as means ± SD

The hemodynamic and respiratory measurements were performed at 1 kHz using an analog‐to‐digital converter (PowerLab; ADInstruments) interfaced using a computer in order to perform off‐line analysis. The diameter and mean blood velocity in the ICA were measured using Doppler ultrasonography and recorded in a computer at 30 Hz using a capture box (DVI2USB 3.0, Epiphan). The diameter and mean blood velocity in the ICA were analyzed at 30 Hz with custom‐designed edge‐detecting and wall‐tracking software (ver. 2.0.1 No. S‐13037, Takei Kiki Kogyo). The ICA SR was calculated using the following formula: 4 × blood velocity/diameter (/s) as in previous studies (Carter et al., 2016; Smith et al., 2019). The ICA flow was calculated as [π × (diameter/2)^2^] × velocity × 60 (ml/min). Cardiopulmonary and cerebrovascular variables were averaged for 2 min of rest (Rest), over the final 4 min of each exercise (Exercise), and for 2 min after stopping the exercise (Recovery). The participants performed each exercise for a total of 12 min. However, we analyzed the cardiopulmonary and cerebrovascular parameters during the final 4 min to compare these parameters between the two different steady‐state exercise conditions because 4 min comprises one cycle of Interval‐Ex, and the work of Interval‐Ex in this period is matched with that of 4 min Cont‐Ex. Moreover, in order to characterize the change in ICA SR, the parameter was averaged every 30 s at rest and from the final 4 min of exercise to the end of recovery (Figure 3).

### 2.6 Statistical analyses

Data were analyzed using the Statistics Package for the Social Sciences (SPSS 26, IBM). Differences between the values were evaluated using a two‐way repeated‐measures analysis of variance (ANOVA, Ex mode × time) followed by Bonferroni's post hoc tests. The effect size was reported with eta‐squared (η^2^) or Hedges' g_av_ using the spreadsheet provided by Lakens (2013). Significance was established at *p* < .05 and denoted statistical significance for all the two‐tailed tests.

## 3 RESULTS

Both the exercise protocols increased the HR, arterial BP, SV, RR, V̇_E_, P_ET_CO_2_, and V̇O_2_ from the baseline rest (Table 1, main effect of time *p* < .001, η^2^ >0.273 for all). The Interval‐Ex has an equivalent work volume of the Cont‐Ex (57.6 kJ); however, increases in the HR and V̇O_2_ during the Interval‐Ex were determined to be larger than those during Cont‐Ex (HR, *p* = .031, Hedges' g_av_ = 0.775; V̇O_2_, *p* = .007, Hedges' g_av_ = 0.844).

**TABLE 1:** Cardiopulmonary and cerebrovascular variables at resting baseline, during exercise, and recovery

	Continuous exercise			Interval exercise			*p*‐value		
	Rest	Exercise	Recovery	Rest	Exercise	Recovery	Ex mode	Time	Interaction
Cardiorespiratory									
HR (beat/min)	74.6 ± 10.6	117.1 ± 15.3*	91.4 ± 17.2^*^, ^†^	71.6 ± 8.1	120.4 ± 16.2^*^, ^#^	96.4 ± 20.3^*^, ^†^, ^#^	.128	<.001	.018
SBP (mmHg)	121.0 ± 15.0	151.2 ± 21.1	127.8 ± 9.2	125.7 ± 13.9	159.3 ± 16.2	134.0 ± 12.5	.106	<.001	.632
DBP (mmHg)	69.7 ± 6.7	78.7 ± 9.0	70.9 ± 9.5	74.3 ± 9.9	83.2 ± 4.9	74.0 ± 4.0	.210	<.001	.626
MAP (mmHg)	86.8 ± 8.6	102.8 ± 12.3	89.9 ± 12.0	91.4 ± 10.2	108.6 ± 6.3	94.0 ± 5.1	.153	<.001	.714
SV (ml)	84.1 ± 16.2	111.3 ± 15.2	93.6 ± 19.2	81.6 ± 13.9	106.0 ± 20.9	93.2 ± 16.8	.409	<.001	.408
RR (breath/min)	14.8 ± 2.9	25.9 ± 7.6	17.1 ± 4.8	15.6 ± 3.0	26.7 ± 6.9	17.8 ± 6.1	.143	<.001	.988
V̇_E_ (L/min)	6.7 ± 1.4	25.8 ± 5.8	15.3 ± 3.4	6.8 ± 1.6	27.0 ± 5.1	17.8 ± 5.9	.002	<.001	.064
P_ET_CO_2_ (mmHg)	39.9 ± 2.4	45.9 ± 4.9*	43.0 ± 4.9^*^, ^†^	39.1 ± 2.5	45.5 ± 3.8*	43.9 ± 5.8*	.810	<.001	.038
V̇O_2_ (ml/min)	178.5 ± 37.0	945.4 ± 131.4*	519.8 ± 77.0^*^, ^†^	174.0 ± 44.2	987.1 ± 116.4^*^, ^#^	622.5 ± 142.8^*^, ^†^, ^#^	<.001	<.001	.002
Cerebrovascular									
ICA diameter (mm)	4.64 ± 0.31	4.65 ± 0.31	4.68 ± 0.33	4.61 ± 0.31	4.60 ± 0.34	4.61 ± 0.33	.016	.483	.393
ICA blood velocity (cm/s)	31.1 ± 3.8	38.1 ± 5.7*	33.7 ± 5.6^†^	31.9 ± 4.9	40.2 ± 7.2*	37.5 ± 8.8^*^, ^†^, ^#^	.030	<.001	.045
ICA blood flow (ml/min)	317.5 ± 53.6	388.9 ± 63.9	348.0 ± 67.8	322.3 ± 63.5	402.6 ± 81.0	377.5 ± 88.5	.151	<.001	.104
ICA conductance (ml/min/mmHg)	3.7 ± 0.7	3.8 ± 0.7	4.0 ± 1.0	3.5 ± 0.6	3.7 ± 0.8	4.0 ± 0.9	.690	.017	.347

Note

Values are means ± SD (n = 11).

Abbreviations: DBP, diastolic pressure; HR, heart rate; ICA, internal carotid artery; MAP, mean arterial pressure; P_ET_CO_2_, end‐tidal partial pressure of carbon dioxide; RR, respiratory rate; SBP, systolic pressure; SV, stroke volume; V̇_E_, ventilation; V̇O_2_; oxygen uptake.

* *p* < .05 different from Rest.

^†^ *p* < .05 different from Exercise.

^#^ *p* < .05 different from Continuous exercise.

No differences were noted in the ICA blood flow and conductance between the different exercise modes (main effect of Ex mode, *p* = .151, η^2^ = 0.011 and *p* = .690, η^2^ = 0.002, respectively); however, an increase in the ICA blood velocity was higher during Interval‐Ex than during Cont‐Ex (*p* = .030, η^2^ = 0.027). Both exercise modes increased ICA SR, while during the final 4 min of exercise and 2 min into recovery, mean ICA SR of the Interval‐Ex was higher than that of the Cont‐Ex (Ex, 351 ± 75 vs. 330 ± 61/s, *p* = .038, Hedges' g_av_ = 0.765; Recovery, 327 ± 86 vs. 290 ± 56/s, *p* = .014, Hedges' g_av_ = 0.814, Figure 3).

## 4 DISCUSSION

This study has identified that Interval‐Ex was associated with elevated ICA SR compared to Cont‐Ex. This finding suggests that as compared to aerobic continuous exercise, aerobic interval exercise could cause higher stimulation to the cerebral vessels in healthy adult men, which may improve cerebral endothelial function, explaining its emergent superior neuroprotective benefits.

This present study demonstrated that subtle changes in exercise intensity increase the cerebral SR (ICA SR); this finding is different from that of the systemic vasculature. In fact, Lyall et al. (2019) measured the vascular SR in the brachial artery during different exercise protocols. In contrast to the cerebral vasculature, the authors reported that an increase in the antegrade SR was equivalent between acute aerobic continuous and interval exercise, although they failed to assess the mean SR. In sum, because acute and chronic increases in the SR are associated with increased endothelial nitric oxide (NO) synthesis and release (Casey et al., 2017; Yan et al., 2007), and NO plays a role in neuroprotective in the cerebral vasculature (Calverley et al., 2020), the findings of this present study and that of Lyall's indicate that aerobic interval exercise may be more neuroprotective in the cerebral vasculature than in the systemic vasculature. Further, exercise‐induced increases in flow‐mediated dilation (FMD) in the systemic vasculature, as an index of endothelial function, were deemed not different between the exercise protocols (Lyall et al., 2019). It is noteworthy that exercise increases the retrograde SR in the systemic vasculature (Lyall et al., 2019); however, retrograde SR is absent in the cerebral vasculature (Rodrigues et al., 2020). In fact, in this present study, the reversal of the ICA blood flow was absent even with the higher workloads during aerobic Interval‐Ex. Moreover, it has been well established that the cerebral vasculature has some specific blood flow regulatory mechanism, such as cerebral autoregulation and cerebral carbon dioxide reactivity (Ogoh, 2008; Ogoh & Ainslie, 2009a, 2009b).

This research background shows that there may be a difference in blood flow regulation and consequently different SR “phenotype (pattern)” between the cerebral and systemic vasculature. One possible mechanism of this dissociation may be due to a different effect of change in the BP on blood flow between cerebral and systemic vasculature. During aerobic Interval‐Ex, the higher workload caused higher ICA SR than that during aerobic Cont‐Ex. It is noteworthy that this higher ICA SR did not decrease acutely and was maintained for a while from the high to low workload or the recovery period (Figure 3). In contrast, in the peripheral artery, aerobic interval exercise increased an oscillation in the SR, indicating that the response of the SR to changes in the exercise strength is quicker than that of the cerebrovascular SR (Lyall et al., 2019; Tremblay et al., 2019). In the cerebral vasculature, likely, this slow SR response to change in the perfusion pressure may be associated with the cerebral autoregulation that compensates the acute change in arterial BP for maintaining cerebral blood flow relatively even during exercise. In contrast, in the systemic vasculature, there are muscle contractions and functional sympatholysis that impact the flow patterns in active skeletal muscle, while thermoregulation and sympathetic vasoconstriction influence the flow patterns in the inactive limbs. Another possible mechanism is associated with that retrograde SR is absent in the cerebral vasculature (Rodrigues et al., 2020). In the systemic vasculature, both the aerobic and resistance exercise have been determined to increase both the antegrade and retrograde SR that typically contribute to the upregulation of the NO pathway (Tinken et al., 2009) and impairment in the endothelial function (Thijssen et al., 2009; Tinken et al., 2009), respectively. However, antegrade SR typically dominates in the cerebral circulation (Rodrigues et al., 2020). It is noteworthy that this different hemodynamic property between the cerebral and systemic vasculatures may highlight the contrasting flow profiles to which these anatomically distinct but functionally integrated vascular beds were exposed (Connolly et al., 2017). Thus, this may also contribute toward the determination of different SRs between the cerebral vasculature and systemic vasculature.

An in vitro study has demonstrated that oscillatory flow profiles with the inherent presence of retrograde SR stimulate a pro‐oxidative‐inflammatory, proatherogenic endothelial cell phenotype (Conway et al., 2010). Moreover, in humans, an acute increase in the retrograde SR has been reported to impair peripheral endothelial function (Thijssen et al., 2009). These findings indicate in the peripheral artery that the increases in the retrograde and the associated oscillatory SR present a detrimental stimulus to the endothelium (Thijssen et al., 2009). However, in contrast to the peripheral vasculature, the reversal of the cerebral blood flow is much smaller or absent; thus, retrograde and oscillatory SRs are likely to be minimal in the cerebral circulation (Rodrigues et al., 2020). In addition to the sustained elevation in the SR, exercise causes transient disturbances in the redox homeostasis in the cells and tissues. Exercise‐induced formation of free radicals, reactive oxygen, and nitrogen species is believed to cause structural tissue damage (Berg et al., 2011). However, emerging evidence suggests that in physiologically controlled, albeit undefined concentrations, they serve as critical signaling molecules that mediate adaptation to the extent cerebral blood flow/SR profile impacts redox homeostasis and subsequent vascular responses (Bailey et al., 2018). However, these suggestions need to be established and require further research.

There are certain limitations of this study. First, we only enrolled men in the present study. The effect of different exercise modes on the cerebral SR may be different in men and women. Second, an effect of different exercise modes on the endothelial function was not identified. The SR may be associated with improved endothelial function (Chatzizisis et al., 2007; Davies, 2009; Thijssen et al., 2009); however, the outcome of cerebrovascular endothelial function to the different SR remains unknown. Thus, further investigations are needed to understand these important issues in detail.

In conclusion, considering the finding in the peripheral artery into consideration, aerobic interval exercise‐induced increases in the SR are more pronounced in the cerebral circulation than in the systemic circulation in healthy adult men. Thus, interval exercise appears to be a more effective exercise for stimulating the cerebral artery and consequently expecting to improve the endothelial function as compared to continuous exercise in healthy adult men. This may prove the fundamental, unifying stimulus that underlies its superior neuroprotective benefits. Thus, we believe that this finding provides important information for building an exercise therapy program that improves arteriosclerosis in the cerebral circulation.

## CONFLICT OF INTEREST

No conflict of interest, financial, or otherwise is declared by the authors.

## AUTHOR CONTRIBUTIONS

Author contributions: S.O. and D.B. conception and design of research; S.O., T.W., K.S., and D.B. performed experiments; S.O., T.W., and E.I. analyzed data; S.O., T.W., E.I., and D.B. interpreted the results of the experiments; T.W. prepared figures; S.O. and D.B. drafted the manuscript; all authors edited and revised the manuscript; all authors approved the final version of the manuscript.
